# Mainstreaming as rhetoric or reality? Gender and global health at the World Bank

**DOI:** 10.12688/wellcomeopenres.13904.2

**Published:** 2018-08-17

**Authors:** Janelle Winters, Genevie Fernandes, Lauren McGivern, Devi Sridhar

**Affiliations:** 1 Global Health Governance Programme, Centre for Global Health Research, Usher Institute of Population Health Sciences, University of Edinburgh Medical School, Edinburgh, UK

**Keywords:** World Bank, gender, global health, mainstreaming, health governance, health financing

## Abstract

**Background: **Over the past decade gender mainstreaming has gained visibility at global health organisations. The World Bank, one of the largest funders of global health activities, released two *World Development Reports *showcasing its gender policies, and recently announced a $1 billion initiative for women’s entrepreneurship. We summarise the development of the Bank’s gender policies and analyse its financing of gender projects in the health sector. This article is intended to provide background for future research on the Bank’s gender and global health portfolio.

**Methods: **First, we constructed a timeline of the Bank’s gender policy development, through a review of published articles, grey literature, and Bank documents and reports. Second, we performed a health-focused analysis of publicly available Bank gender project databases, to track its financing of health sector projects with a gender ‘theme’ from 1985-2017.

**Results: **The Bank’s gender policy developed through four major phases from 1972-2017: ‘women in development’ (WID), institutionalisation of WID, gender mainstreaming, and gender equality through ‘smart economics’. In the more inclusive Bank project database, projects with a gender theme comprised between 1.3% (1985-1989) and 6.2% (2010-2016) of all Bank commitments.  Most funding targeted middle-income countries and particular health themes, including communicable diseases and health systems. Major gender-related trust funds were absent from both databases. The Bank reports that 98% of its lending is ‘gender informed’, which indicates that the gender theme used in its publicly available project databases is poorly aligned with its criteria for gender informed projects.

**Conclusion: **The Bank focused most of its health sector gender projects on women’s and girls’ issues. It is increasingly embracing private sector financing of its gender activities, which may impact its poverty alleviation agenda. Measuring the success of gender mainstreaming in global health will require the Bank to release more information about its gender indicators and projects.

## Introduction

Over the last decade, particularly since the launch of the sustainable development goals (SDGs) in 2015, gender has become increasingly visible within the global health community. The Bill and Melinda Gates Foundation selected gender as a ‘grand challenge’ for the first time in 2014 ^1^ and pledged $80 million to close gender data gaps in 2016. This announcement was followed by a call to action from the ‘Women in Global Health’ initiative ^2^, which in turn was instrumental in lobbying World Health Organization (WHO) Director General Tedros Adahanom Ghrebreyesus to appoint 60% women to the WHO’s leadership team for the first time in its – and in fact in any UN institution’s – history (reports here and here) ^2^.

Yet, such widespread attention has not come without controversy. The Ebola and Zika epidemics, in particular, brought issues of ‘gender blindness’ to the forefront, as women’s voices were often underrepresented in planning and response activities, in spite of the fact that they were disproportionately affected by the outbreaks ^3, 4^. Researchers and policymakers have questioned how major international development organisations define and frame gender ^5, 6^. A dominant critique is that gender is often seen through the lens of women’s and girls’ empowerment, particularly through education (i.e. Millennium Development Goal 3) ^7, 8^. This may exclude men and members of the LGBTI community who have the highest burden of disease in some health contexts ^5, 9, 10^. Others have argued that the gender equality rhetoric does not match reality, as evidenced by few women in leadership and decision-making positions at global health organisations ^2, 11^. Finally, scholars worry that the rising involvement of the private sector in health (e.g. through global public-private partnerships) could link corporate profit with gender equality ^12– 14^.

Such private investments in gender programming have come into vogue since the late 2000s, the newest of which is the World Bank’s Women in Entrepreneurs Finance Initiative (We-Fi). In 2017, G20 leaders pledged approximately $1 billion into this trust fund (a financing vehicle for voluntary contributions, of which the Bank serves as trustee) ^15^, which will be implemented jointly by the traditional World Bank (the International Bank for Reconstruction and Development and International Development Association) and its private financing arm, the International Finance Corporation (IFC). The World Bank is arguably the most influential institution in global health, both ideologically and financially ^16^. Although its historical links to neoliberalism ^17^ have raised questions about its commitment to poverty alleviation and women’s empowerment ^18^, the Bank publicly advocated for gender equity and mainstreaming through its heavily cited 2002 and 2012 *World Development Reports*. Indeed, the Bank has pointed to its high level of success in mainstreaming gender, especially in the health sector. From 1988–1999, it estimated that 89% of Health, Nutrition and Population (HNP) projects contained gender considerations ^19, 20^, compared to 38% of Bank projects across all sectors, and in 2013, 97% of all Bank projects were deemed ‘gender informed’.

**Figure 1.**
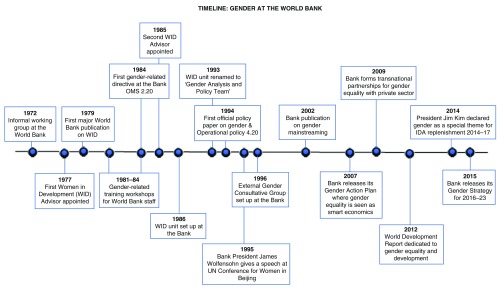
Timeline of gender policy development at the World Bank. The World Bank introduced gender policy for development in the 1970s through a ‘women in development’ approach, increasingly institutionalized and mainstreamed its policy in the 1980s–1990s, and began a ‘smart economics’ to gender equality approach in the mid-1990s.

**Figure 2.**
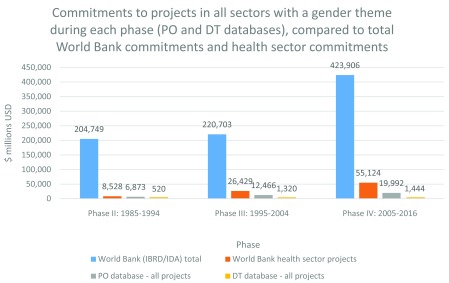
Commitments to projects with a gender theme during each historic phase, relative to total World Bank commitments and health sector commitments. Gender projects across all sectors increased in both absolute and relative terms between Phase II (1985–1994) and Phase IV (2005–2009), according to data analysed from the Bank’s project databases. IBRD = International Bank for Reconstruction and Develompment; IDA = International Development Association; PO = Projects & Operations; DT = Development Topics.

However, does the rhetoric of the Bank’s work in gender match the reality of its operations and lending portfolio, in the context of global health? This paper provides a scoping review of the Bank’s gender framework since the 1970s and its corresponding financial flows to health sector projects with a gender theme since 1985, using publicly available sources. It is intended as a roadmap for future research on gender, mainstreaming indicators, and global health at the Bank. Using Bank reports and secondary literature, we first explore the Bank’s conceptualisation of and policies for gender over time. In doing so, we identify four phases of the Bank’s gender approach ( Figure 1): the launch of ‘women in development’ (WID) (1972–1984), the institutionalisation of WID at the Bank (1985–1994), gender mainstreaming (1995–2004), and gender equality through ‘smart economics’ (2005–2017). We illustrate Bank commitments to projects with a gender theme during three of these phases ( Figure 2). We then position global health financing within this gender framework, using two major Bank project databases and the Bank’s Monitoring Gender Mainstreaming financial database. By tracking Bank health sector projects with a gender theme in these databases, we provide a snapshot of trends in the Bank’s support of gender in global health and highlight significant transparency issues.

## Methods

This paper relies on two major data sources. First, we used published articles and grey literature reports to construct a timeline of the framing and operationalising of gender at the World Bank. Second, we extracted financial data from publicly available gender project databases, and analysed this data for the health sector.

PubMed and Scopus online databases were systematically searched for relevant published articles using the key words ‘World Bank’ and ‘gender’. We selected articles that presented any information on gender-related activities, events, guidelines, and policies at the World Bank and were published in English in a peer-reviewed journal. A total of 307 search results were reviewed for the aforementioned criteria, and 20 were included in this analysis. Additional peer-reviewed publications were identified through the reference lists of these 20 articles. Finally, we identified and analysed key publications on gender by the World Bank, its Operations Evaluation Department, and its Independent Evaluation Group.

**Figure 3.**
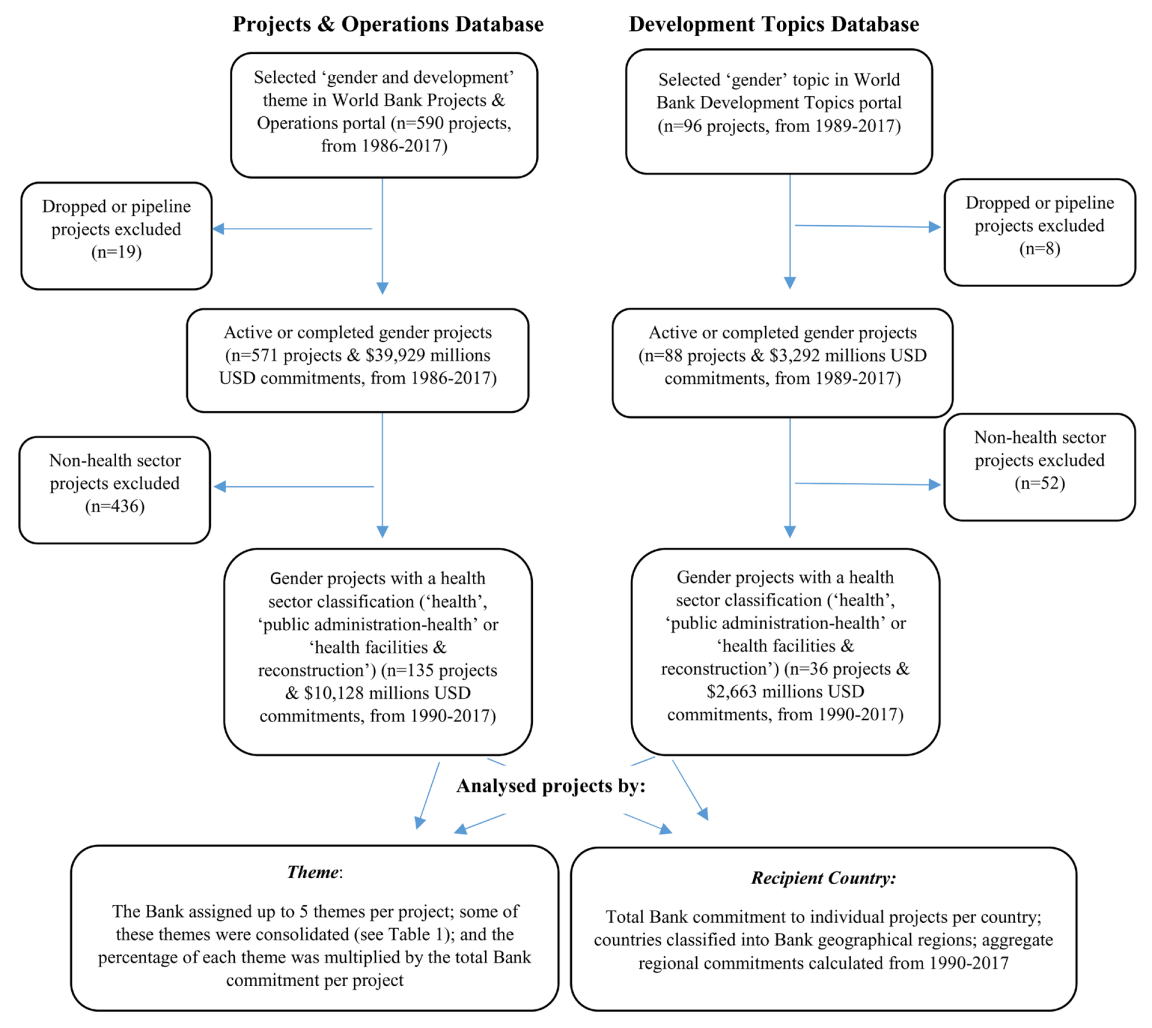
Gender and health analysis framework and inclusion criteria for World Bank project databases. All active and completed World Bank gender projects from 1985 - July 1, 2017 were analysed using two World Bank databases, and Bank commitments were tracked for health sector projects.

Data on World Bank financing of projects with a gender component are available publicly through the Bank’s ‘Projects & Operations’ (PO) and ‘Development Topics’ (DT) databases. Both databases include projects with gender focuses from 1985–2017 and allow projects to be searched by sector and theme, but they do not include identical projects. In order to understand the Bank’s reported funding for gender projects in the health sector, we therefore exported project data from both databases (as of July 1, 2017). Figure 3 provides a summary of the inclusion/exclusion criteria and analysis framework. We classified projects as ‘gender projects’ if they had a gender theme listed (see definition in Table 1), regardless of the percentage given for this theme. The Bank lists up to five themes for each project, and the gender theme percentage for projects varied from 5% to 100%. Theoretically, therefore, the gender theme should capture projects with even a minor gender component.

**Table 1.** Definitions of World Bank health and gender themes. *Adapted from World Bank Theme Taxonomy & Definitions, July 2016* ^21^.

Theme	Definition
**Child health**	Activities aimed to improve the health status of children and to reduce child morbidity and mortality.
**Gender**	For the purposes of coding, the theme encompasses World Bank Group activities that – irrespective of sector – address and/or close gaps between males and females and other gaps that may be identified at the Country Partnership Framework at the country level.
**Communicable** **diseases**	*HIV/AIDS –* Programmes that increase access to HIV/AIDS prevention, treatment, care and support services. *Tuberculosis –* Activities aimed at the prevention, diagnosis and/or treatment of tuberculosis. *Malaria –* Activities aimed at the prevention, diagnosis, control and/or treatment of malaria.
**Health system** **performance**	Programmes and policies which aim to bring about improvements in the management, financing and overall functioning of health systems.
**Injuries & non-** **communicable** **diseases**	Activities aimed to reduce morbidity and premature mortality from cardiovascular disease, hypertension, cerebrovascular disease, peripheral vascular disease, cancer, chronic obstructive pulmonary disease, asthma, diabetes, mental illness (including depression, post-traumatic stress disorder, suicide, psychosis, alcohol and drug abuse), and other non-infectious, chronic conditions such as arthritis and osteoporosis. This theme also includes preventable injuries (excluding road/traffic accidents).
**Nutrition & food** **safety**	Programmes that include objectives and specific activities related to improving nutritional status or food security at the household level.
**Population &** **reproductive health**	Activities to improve reproductive health and reduce maternal morbidity and mortality.

For each database, projects with a health sector classification were selected for further analysis, and the absolute Bank commitments to gender projects in the health sector were calculated. Although some projects took place over multiple years, the Bank releases funding data by the project’s approval date (PO database) or starting year (DT database), and all commitments were assigned to this year. We then disaggregated all health sector project commitments by theme (health and gender themes are defined in Table 1), to determine the relative World Bank funding for health themes each year. To facilitate comparison of health themes, some Bank themes were combined (see Table 2). Finally, we determined the total commitments for gender projects in the health sector for each recipient country and geographical region from 1990–2017. We also compared the scope of both databases by identifying the number of identical projects that they included.

**Table 2.** Themes used for analysis of gender projects in both gender project databases. *A total of 49 themes were used by the Bank to categorize the gender projects, so some themes were combined into broader theme categories for analysis.
*

Compiled theme used for analysis	Corresponding World Bank theme(s) given to projects
**Communicable diseases**	HIV/AIDS Communicable diseases Malaria Tuberculosis
**Child health**	Child health
**Nutrition & food security**	Nutrition & food security
**Gender**	Gender
**Health system performance**	Health system performance
**Population & reproductive health**	Population & reproductive health
**Injuries & non-communicable diseases**	Injuries & non-communicable diseases
**Environment**	Pollution management & environmental health Environmental policies & institutions Water resource management National disaster management
**Education**	Education for all
**Social development**	Other social protection & risk management Social analysis & monitoring Social risk mitigation Social protection Other social development Social safety nets Social inclusion
**Rural services**	Rural services & infrastructure Rural markets Other rural development Rural policies & institutions
**Participation & civic engagement**	Participation & civic engagement
**Conflict preparation & reconstruction**	Conflict prevention & post-conflict reconstruction
**Private sector development**	Micro, small & medium enterprise support Infrastructure services for private sector development Other private sector development
**Public sector development**	Municipal governance & institution building Other economic management Improving labour markets State-owned enterprise restructuring & privatization Macroeconomic management Law reform Tax policy & administration Decentralization Administrative & civil service reform Regional integration Other public service governance Public expenditure, financial management & procurement
**Poverty & urban development**	Other human development Vulnerability & assessment monitoring Urban services & housing for the poor Poverty strategy, analysis & monitoring Other urban development
**Indigenous peoples**	Indigenous peoples

Additionally, the Bank releases data about its gender indicator in a publicly available database, ‘ Monitoring Gender Mainstreaming in World Bank Lending Operations’, through its World Bank Group Finances platform. This database is less useful for understanding historical funding for gender at the project level, because it only includes projects from 2009 to 2016. It is not searchable by sector, but does include a category for projects in the Health, Nutrition and Population (HNP) global practice, from 2013 onwards. Unlike the PO and DT databases, this database includes gender indicator scores, which classify a project based on whether gender is considered at its analysis, actions, or monitoring and evaluation dimensions (a score of 0 means a project is not gender informed, and 1–3 that it is gender informed). We extracted all HNP projects from the Monitoring Gender Mainstreaming database, and determined the Bank’s aggregate commitments to gender informed projects from 2013–2016. We then determined how well the gender informed indicator corresponded with the gender theme for HNP projects, by comparing gender theme classifications for each project (yes, no, or blank/no entry given). Finally, we looked for overlaps between this database and the PO and DT databases, to see how inclusive the PO and DT databases were since 2013.

In order to position the Bank’s health sector gender project financing within wider development assistance for health trends, we compared PO and DT gender project commitments to those reported by the Organisation for Economic Co-operation and Development OECD). The Institute for Health Metrics and Evaluation (IHME)’s development assistance for health data does not include a gender marker, so the OECD Development Assistance Committee’s Creditor Reporting System ( DAC-CRS) database provides the most comprehensive publicly available data on gender considerations within development assistance for health. First, gender projects funded by all OECD donors were targeted using the ‘gender equality and women’s empowerment’ policy marker, which tracks projects with gender objectives from 2002–2015. Second, these gender projects were sorted by target geographical region, health sector category, and year. We recorded all OECD CRS funding in constant 2015 USD commitments, and included projects with gender as a ‘principal’ or ‘significant’ objective (see OECD gender equality policy marker handbook). For comparative analyses, OECD health sector themes were combined: ‘communicable diseases’ includes infectious disease control, malaria control, and tuberculosis control themes, while ‘health systems’ includes basic health care, basic health infrastructure, and health personnel themes.

## Results

### Timeline: The governance of gender at the World Bank, 1972–2017

***Women in development (WID): 1972 to 1984***. The first phase of gender at the World Bank (1972–1984) was characterised by growing theoretical arguments for women’s importance to development, but little institutional impetus for their implementation. Initially, the Bank’s involvement was limited to an informal working group of Bank staff, formed in 1972, which discussed the concerns of women in the institution ^22^. The United Nations (UN) declaration of the International Women’s Year in 1975 prompted the Bank to publish a booklet, *Integrating Women in Development*, which described measures to reach women through Bank projects. Around the same time, it coined the term ‘Women in Development’ (WID), to promote development activities that benefitted women ^22, 23^.

Owing to internal staff pressure, the Bank appointed its first WID advisor – a UN official and sociologist – in 1977 ^22^. The advisor was tasked with increasing Bank staff and borrowers’ understanding of how women affected and were affected by Bank projects ^24^. As a result of this appointment, from 1979–1985, the Bank published its first major gender publication ^25, 26^, completed thirty-five case studies of gender issues in Bank projects, and held five gender-related workshops for staff ^22^. While these activities provided legitimacy for gender issues at the Bank, they were of limited scope and met with scepticism by many Bank staff. For instance, Bank Presidents Robert McNamara and Alden Clausen focused primarily on reproduction and population control in their speeches ^27, 28^, and the Bank’s first gender-related directive (1984) – which called on staff to consider women in the project cycle – only applied to projects in which women were considered important beneficiaries or recipients ^29^. In this phase, women’s issues received sporadic attention in the Bank’s lending decisions ^30^, and the WID advisor’s budget never exceeded $90,000 ^22, 31^.

***Institutionalising WID in the Bank: 1985 to 1994***. During the second phase (1985–1994), theories of human capital were integrated into the Bank’s gender framework and gender was broadly institutionalised. A series of changes to gender policies occurred in fast succession; a senior Bank economist was appointed the new WID advisor in 1985 ^30^, a WID unit was established in 1987 ^20^, and Bank President Barber Conable designated gender as one of four formal areas of special interest the same year. This top-down support of the WID framework gave earlier gender directives ‘teeth’, as managers were required to show that they addressed women’s issues in their portfolios. The WID budget grew to $2.5 million in 1992, and with it the WID team expanded both at Bank headquarters and through regional coordinators ^22^.

The Bank’s interest in the economic gains of investing in women led to a series of gender studies in the late 1980s and early 1990s ^32^, culminating in its first official policy paper on gender ^33^ and new ‘Gender and Development’ approach ^6^. This approach argued that women should be analysed in relation to men, rather than independently. It underscored the human capital argument for investing in women: financing empowerment activities could increase productivity, promote efficient use of resources, lead to social returns (like family planning and child survival), and reduce poverty ^33, 34^. A new policy (OP 4.20) reinforced the Bank’s commitment to consider gender during the project cycle, through its Country Assessment Strategies. In line with its evolving conceptualisation of gender, the Bank renamed the WID division the ‘Gender Analysis and Policy Team’ in 1994 ^22^.

***Gender mainstreaming: 1995 to 2004***. In the third phase (1995–2004), the Bank focused on mainstreaming gender in its lending operations and increasingly embraced the concept of gender equality. Gender mainstreaming became visible at the Bank in 1995 when Bank President James Wolfensohn gave a speech at the Fourth World Conference on Women in Beijing ^20^. Subsequently, a campaign called ‘Women’s Eyes on the World Bank’ called for increased participation of women in the Bank’s policies and programmes ^35^. In response to this push for more inclusive gender policies, the Bank created an External Gender Consultative Group, which included representatives of civil society, non-governmental, academic, and political organisations ^36^. It also established a Gender and Development Board within the Bank’s Poverty Reduction and Economic Management Network in 1997, to monitor and report on the status of policy implementation ^20^. Finally, the Bank developed tools to measure gender’s role in development, including sex-disaggregated statistics, gender impact assessments, and gender monitoring procedures ^37– 39^.

Gender mainstreaming was justified by its role in economic growth, poverty reduction, development effectiveness, and promoting gender equality ^40^. As part of its mainstreaming efforts, the Bank expanded its existing operational policy on gender consideration, which covered social sector programmes, to include all foreign direct investment programmes ^41^. Ultimately, between 1995 and 2001, the proportion of projects that included some consideration of gender issues in their design almost doubled, to nearly 40% ^42^.

***Gender equality as smart economics: 2005 to 2017***. In the most recent phase, the Bank promoted ‘smart economics’ and showcased its approach to gender within the international community. A dominant theme in this phase is the tension between framing gender equality as a human right and an agent for economic growth. The Bank’s 2006 *World Development Report* argued that gender equality is important both in its own right and as an instrument for faster economic growth, particularly in the Global South ^43^. Its subsequent *Gender Equality as Smart Economics* report (2007) dubbed such investment in women and girls for development goals ‘smart economics’, and largely omitted the concept of gender equality as a human right ^44, 45^. Finally, in its seminal 2012 *World Development Report*, the Bank reasserted the importance of gender equality as an objective in its own right, while also promoting smart economics ^46^.

At the same time that these reports placed the Bank’s framework for gender on the international stage, President Jim Yong Kim and Bank leaders reinforced the importance of gender within the institution. For instance, gender was declared a ‘cross-cutting solution’ and was used as a ‘special theme’ in the successful 2014–2017 replenishment of the Bank’s concessional lending arm, the International Development Association (IDA) ^18, 47^. Over the past decade, the Bank’s mechanisms for achieving these gender equality and development goals have increasingly involved the private sector. For instance, the Bank began to form transnational partnerships for gender equality with private sector organisations in the late 2000s ^48^, and Bank President Robert Zoellick emphasized the importance of ‘investing in women’ at the 2009 Global Private Sector Leaders Forum, which included corporate giants like Nike, ExxonMobil, and Goldman Sachs ^49^. The Bank’s latest gender strategy (2016–2023) includes human rights and aspects of smart economics, but expands conceptions of gender equality to include enhancing women’s voice and agency ^50^.

## The World Bank’s lending portfolio

### Financing for health sector projects with a gender theme or consideration

The Projects & Operations (PO) database provided more comprehensive coverage of Bank commitments to gender projects than the Development Topics (DT) database. According to the PO database, the Bank committed $39.3 billion to gender-related projects from 1985–July 2017, which ranged from 1.25% to 6.16% of total IBRD and IDA commitments. The DT database recorded $3.3 billion in gender-related projects during the same years, or between 0.02% and 0.4% of the Bank’s commitments from 1985–2017 ( Table 3) ^16^.

**Table 3.** Gender funding at the World Bank relative to total funding for IBRD/IDA projects and Health, Nutrition and Population (HNP) sector projects ^20, 51^. IBRD = International Bank for Reconstruction and Development; IDA = International Development Association; HNP = Health, Nutrition and Population; PO = Projects & Operations; DT = Development Topics.

Commitments ($ millions USD)	1985–1989	1990–1994	1995–1999	2000–2004	2005–2009	2010–2016
**World Bank (IBRD/IDA) total**	92,770	111,979	120,418	100,285	162,580	261,326
**World Bank HNP new commitments**	1,551	5,305	8,263	6,796	8,093	18,383
**World Bank health sector project commitments**	1,386	7,142	10,925	15,544	20,074	35,051
**Gender PO database – all** **projects**	1,163	5,710	6,450	6,016	3,891	16,101
**% of IBRD/IDA total**	1.3%	5.1%	5.4%	6.0%	2.4%	6.2%
**Gender DT database – all projects**	20	500	305	1,015	407	1,037
**% of IBRD/IDA total**	0.02%	0.5%	0.3%	1.0%	0.2%	0.4%
**Gender PO database – health sector**	0	1,105	2,510	1,849	2,208	3,908
**% of health sector projects total**	0.0%	15.5%	23.0%	11.9%	11.0%	11.2%
**Gender DT database – health sector**	0	423	292.7	886	389	579
**% of health sector projects total**	0.0%	5.9%	2.7%	5.7%	1.9%	1.7%
**Health % of all gender projects - PO database**	0.0%	19.4%	38.9%	30.7%	56.8%	24.3%
**Health % of all gender projects - DT database**	0.0%	84.6%	96.0%	87.3%	95.6%	55.8%

**Figure 4.**
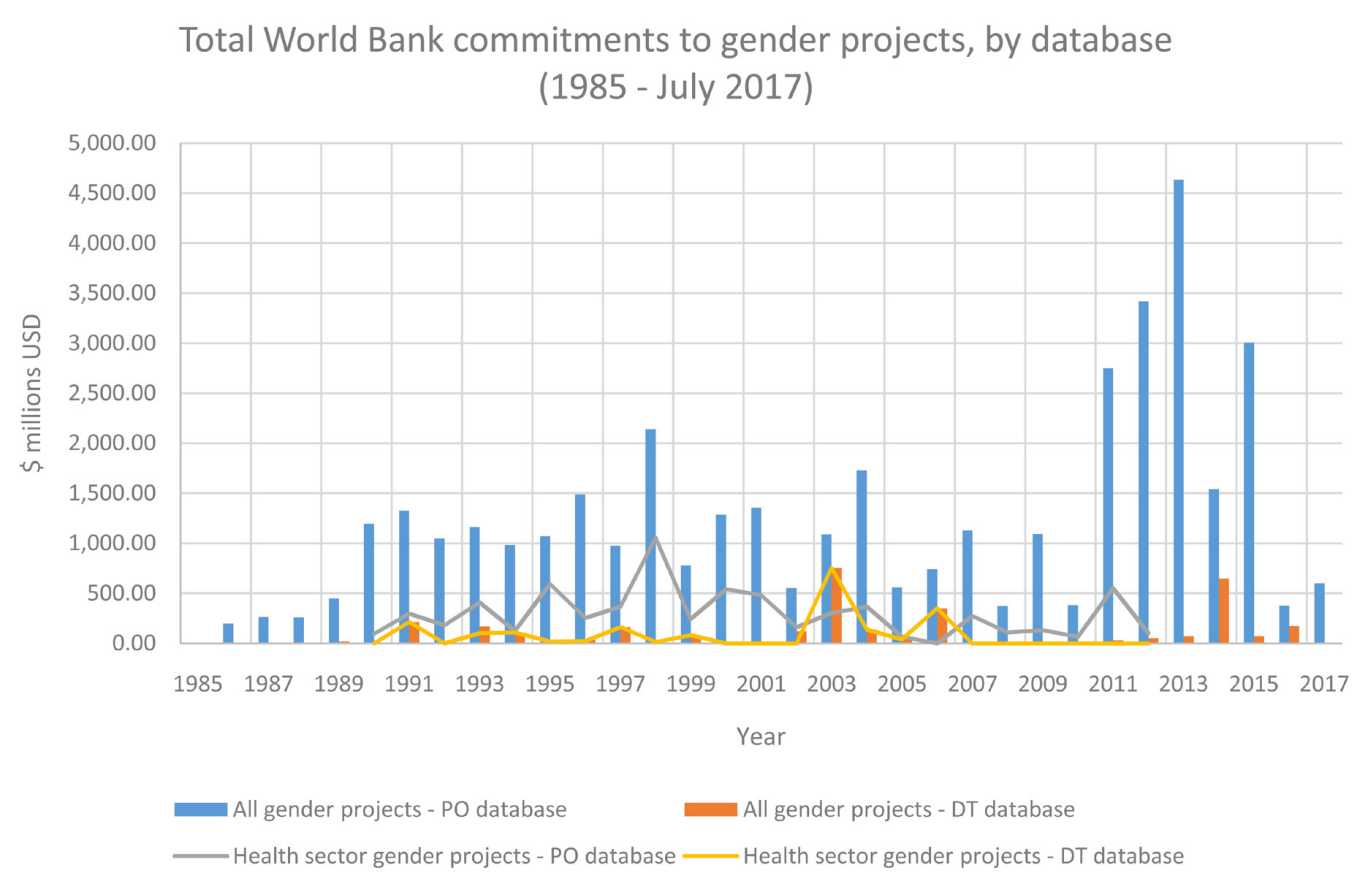
World Bank funding for projects with a gender theme in all sectors and in the health sectors, by database, from 1985-July 2017. Both databases show an increase in the World Bank’s commitments to gender projects since 1985, but this increase is volatile and peak gender financing for the health sector is inconsistent. PO = Projects & Operations; DT = Development Topics.

**Figure 5.**
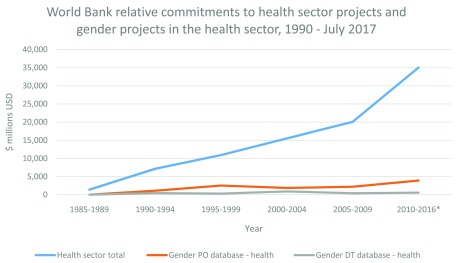
Funding for projects with a gender theme in the health sector compared to all health sector (Health, Nutrition, and Population) projects at the World Bank, from 1990–2017. The World Bank’s health sector portfolio has grown faster than financing for gender projects in the health sector. According to both analysed databases, health sector projects with gender components made-up less than 12% of total HNP commitments from 2010-2016. PO = Projects & Operations; DT = Development Topics.

In both databases, the health sector represented a high percentage of the Bank’s total commitments to gender projects ( Figure 4). The percentage of the health sector within all gender projects peaked at 56.8% in 2005–2009 for the PO dataset, and at 96.0% in 1995–1999 for the DT dataset. However, while many gender projects were in the health sector, they consistently formed only a small part of the Bank’s total funding for health sector projects ( Figure 5). The PO database’s commitments to gender projects in the health sector formed a maximum of 23.0% of the Bank’s commitments to health sector projects (in 1995–2009), and only approximately 11% of health sector commitments since 2005 ( Table 3). Despite the fact that both databases included projects with a gender theme, they only had four identical projects. This indicates that even the more inclusive PO database may be missing a significant number of health sector projects with a gender theme.

The Monitoring Gender Mainstreaming database included 89 HNP projects totalling $8.61 billion in World Bank commitments, from FY2014 to FY2017 (2013–2016). The Bank reports that its commitments to all new HNP projects from 2013–2016 was $9.42 billion, meaning that approximately 91.4% of HNP lending during this period was gender informed. The vast majority, 85.4%, of gender informed HNP projects in the database had a gender informed indicator score of 3 (i.e. gender was considered at the planning phases of project analysis, actions, *and* monitoring and evaluation), while only one project had a score of 1 (i.e. gender was considered at the planning phase of only one of these project dimensions). However, the gender informed indicator was poorly correlated with the gender theme. Only three of the 89 gender informed projects were classified as having a gender theme; thirteen projects with a highly gender informed score of 3 were classified as having no gender theme; and 39 gender informed projects had no marking (neither a yes nor a no) for the gender theme category. The database did not include a category for project sector, and more than half of all of the 2645 projects listed did not include a global practice classification. It was therefore not possible to easily track HNP gender informed projects before 2013, and it is possible that our analysis missed some wider health and social services sector projects after 2013.

### Health themes & recipient countries for gender projects

**Figure 6.**
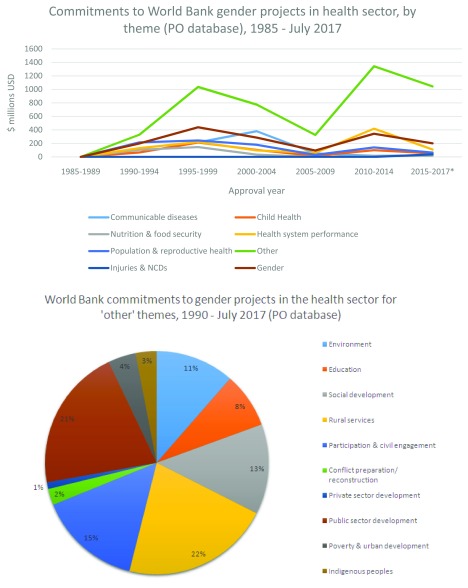
Total World Bank funding for projects with a gender theme in the health sector for the PO database, by theme. The World Bank’s commitments to gender projects in the health sector included many themes, and the relative financing for communicable diseases, health system performance, population and reproductive health, and child health varied over time. This data was obtained from the more comprehensive Projects & Operations (PO) database. NCD = non-communicable disease.

**Figure 7.**
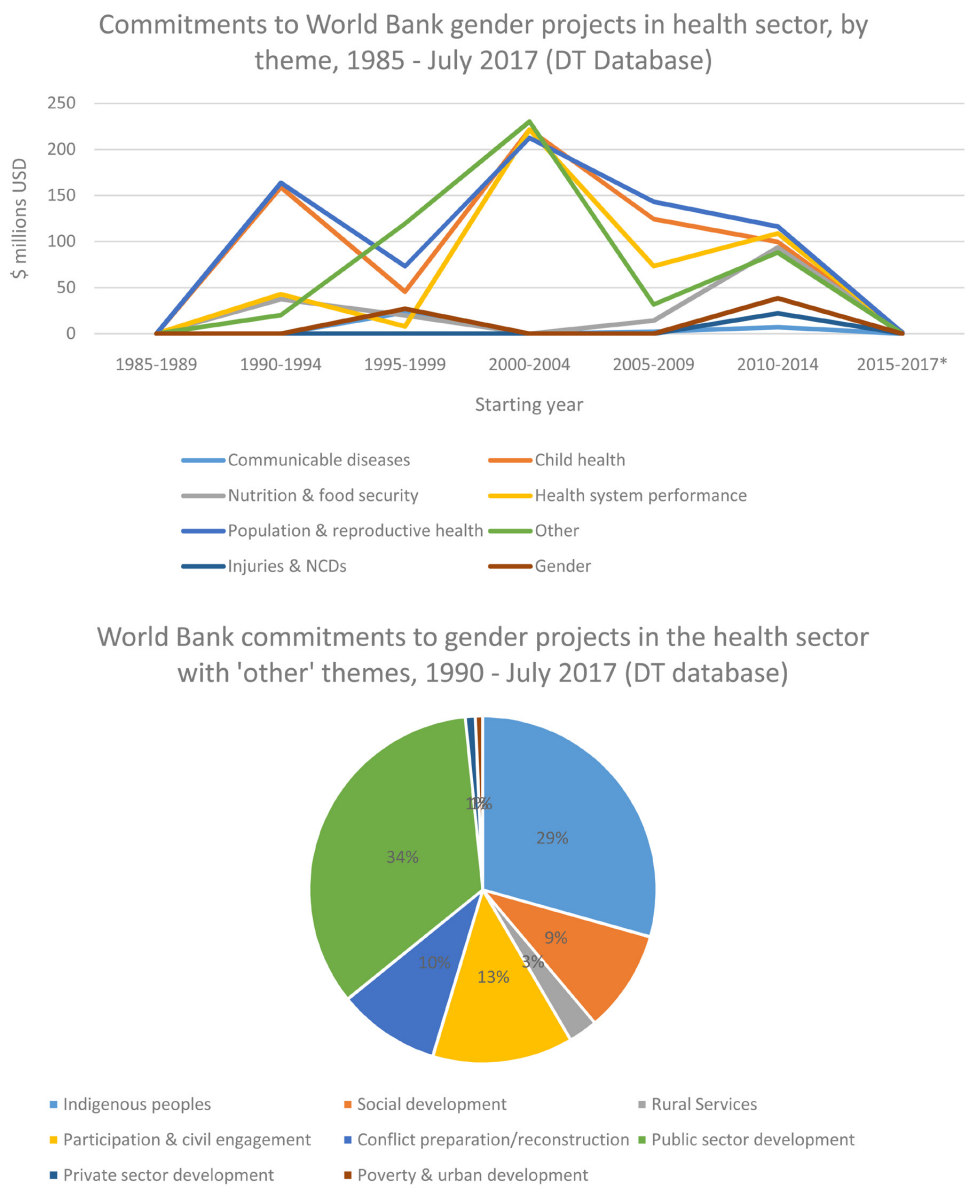
Total World Bank funding for projects with a gender theme in the health sector for the DT database, by theme. The Development Topics (DT) database included similar health sector themes to the more inclusive Projects & Operations (PO) database, and included less projects with a communicable disease theme. NCD = non-communicable disease.

For projects with a gender theme in the health sector, particular health themes were emphasized over others ( Figure 6 and Figure 7). Definitions of the major health themes are given in Table 2. In the PO database, communicable disease and health system performance themes received the highest commitments. Funding for gender projects with a communicable disease theme peaked in 2000–2004 and have since declined, while funding for health system performance remained relatively steady from 1990–2015 and peaked in 2010–2014. Population and reproductive health and child health projects with a gender theme received relatively less funding from 1990–2015, and only one project targeted injuries and non-communicable diseases during this period. The DT database similarly included only one project for injuries and non-communicable diseases from 1985–2017. However, the relative importance of population and reproductive health, child health, communicable disease, and health system performance was different in the DT than the PO database ( Figure 6 and Figure 7). The DT database lacked many health projects included in the PO database, and particularly omitted communicable disease projects.

**Figure 8.**
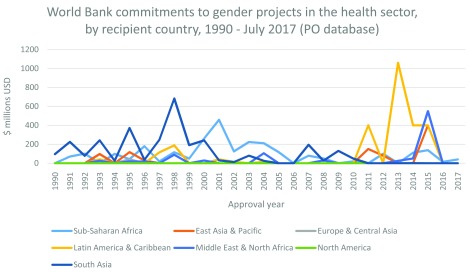
World Bank commitments to projects with a gender theme in the health sector, by recipient country region, 1990–2017 (PO database). The recipient regions for World Bank-funded gender projects in the health sector showed significant volatility from 1990–2017, with Latin America & the Caribbean and South Asia receiving the highest commitments. This data was obtained from the more comprehensive Projects & Operations (PO) database.

**Figure 9.**
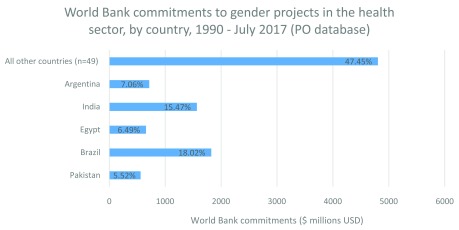
Proportional funding by country for World Bank commitments to projects with a gender theme in the health sector, 1990–2017 (PO database). Five countries received over half of all Bank commitments for gender projects in the health sector from 1990–2017. This data was obtained from the more comprehensive Projects & Operations (PO) database.

Bank commitments to gender projects in the health sector were given inconsistently to countries and geographic regions over time ( Figure 8). For instance, in the PO dataset, low-income countries in Sub-Saharan Africa received funding for gender projects in the health sector most years from 1990–2017. However, this funding was typically in small commitments to many different countries. In contrast, the large commitments for health sector projects were given to five countries: Brazil, India, Argentina, Pakistan, and Egypt. These five lower- and upper- middle income countries collectively received nearly half of all Bank commitments to gender projects in the health sector from 1990–2017 ( Figure 9). The less inclusive DT database revealed an even sharper preference for funding middle income countries, with Argentina receiving over half (52%) of all health sector and gender funding. Europe and Central Asia and North America received no or extremely little funding in both databases, and East Asia and the Pacific and the MENA regions collectively received between 7% and 21% of the commitments from 1990–2017.

The DT database also included some projects financed by trust funds, to which donors (but not the Bank) made contributions. Trust funds at the Bank are sometimes called ‘multi-bi’ or ‘extra-budgetary’ aid, because they use voluntarily contributed funds from specific donors to finance activities ^15^. From 2002–2017, $89.3 million was invested in gender projects by donors through recipient-executed trust funds (Bank trust funds that are executed directly by a country or organisation), of which $17.6 million was for health projects. These health projects were primarily for maternal and child health programmes in low income countries, through the Japan Social Development Fund. Some donor commitments ($5.7 million) to the Bank’s Gender Trust Funds (GENTF) programme were included in this dataset, but they were not for the health sector.

**Figure 10.**
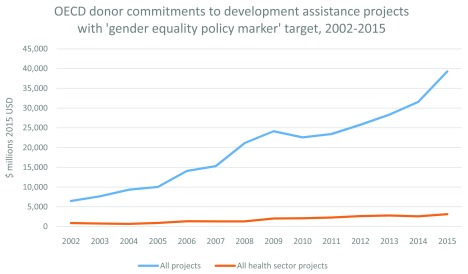
OECD donor development assistance for gender projects across all sectors and in the health sector, from 2002–2015 (DAC-CRS). Organisation for Economic Co-operation and Development (OECD) donor commitments to development assistance projects with a ‘principal’ or ‘significant’ gender equality policy marker have increased relatively less for health sector projects than all projects since 2002. This data was obtained from the Development Assistance Committee Creditor Reporting System (DAC-CRS) database.

***Wider OECD financing for gender projects in the health sector***. To contextualize the Bank’s gender commitments within the larger aid landscape, we tracked OECD donor development assistance commitments to the health sector, using the ‘gender equality policy marker’, from 2002–2015. This policy marker is based on a gender mainstreaming checklist, which requires donors to state whether a project has a gender dimension or impact, and whether this is at a principal or significant level. OECD donor contributions to all gender projects increased from $6.5 billion in 2002 to $39.3 billion in 2015. Health sector projects averaged about 10% of all OECD commitments to gender projects from 2002–2015 ( Figure 10). Based on our PO database commitments, the World Bank therefore contributed approximately 10% of the total development assistance for gender projects from 2002–2013, but the Bank’s relative commitments dropped after 2013.

**Figure 11.**
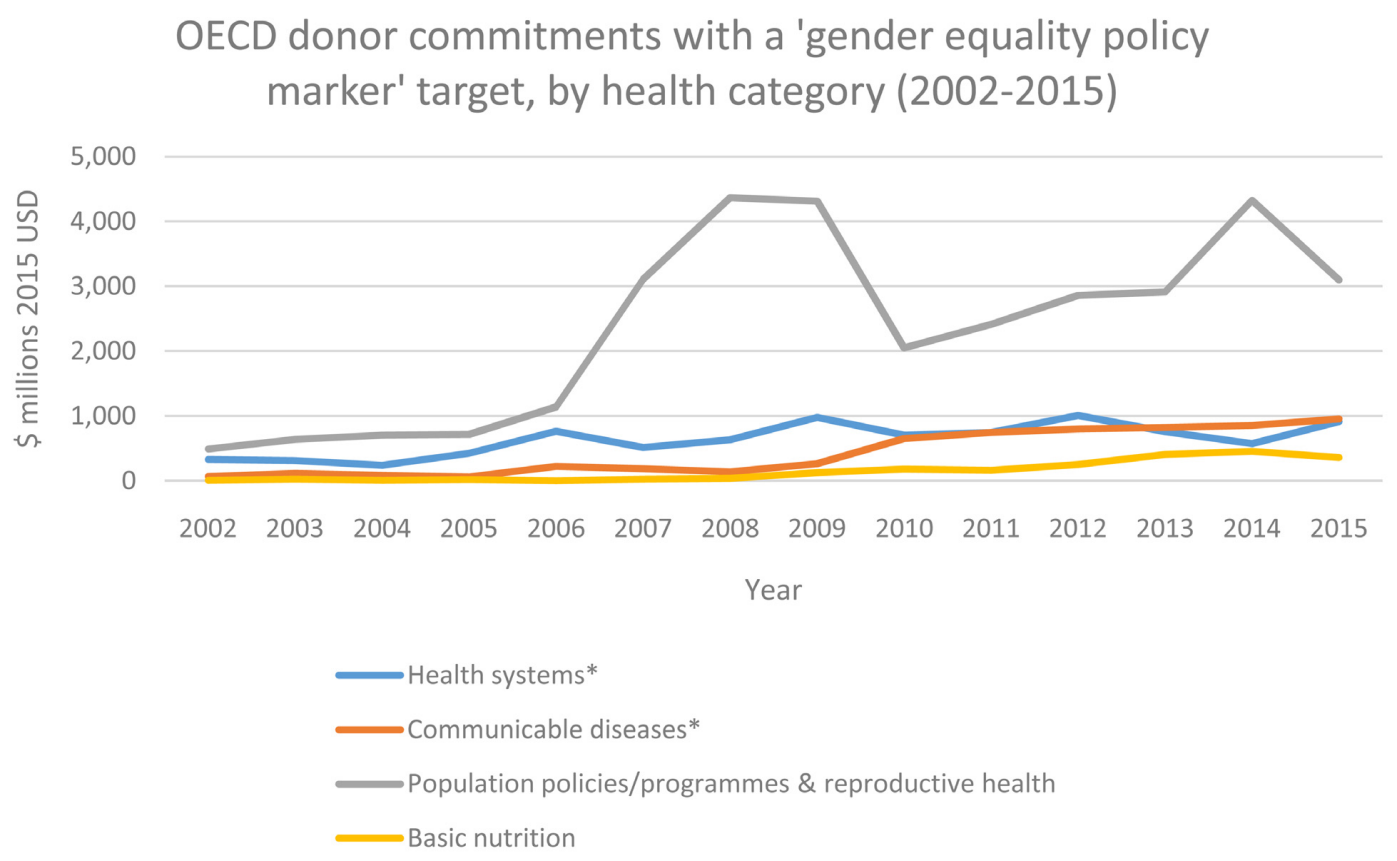
Total OECD donor commitments to gender projects in the health sector from 2002–2015, by health category. * *Health systems includes basic health care, basic health infrastructure, and health personnel. Communicable diseases includes infectious disease control, malaria control, and tuberculosis control.* Organisation for Economic Co-operation and Development (OECD) donor commitments to development assistance for health projects with a principal or significant gender target emphasized population and reproductive health from 2002–2015.

**Figure 12.**
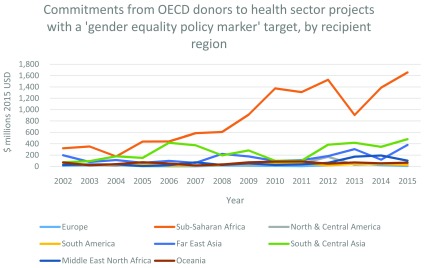
Development assistance for health from all OECD donors for health sector projects with a ‘gender equality policy marker’ target (2002–2015), by recipient region. Organisation for Economic Co-operation and Development (OECD) donor commitments to development assistance for health projects with a principal or significant gender target predominately financed projects in Sub-Saharan Africa from 2002–2015.

The Bank emphasized gender projects in the health sector relatively more than OECD donors; according to the PO database, Bank health sector commitments averaged 37.3% of all gender commitments from 2000–2017 ( Table 3), while those of OECD donors were 11.0% from 2002–2015. Within these health sector commitments, OECD donors prioritized reproductive policies and population health projects more than the Bank ( Figure 11), while the Bank prioritized health systems and communicable disease gender projects more than OECD donors ( Figure 5 and Figure 11). Unlike the Bank, a high proportion (58%) of OECD donor commitments to gender projects in the health sector from 2002–2015 targeted low-income countries in Sub-Saharan Africa ( Figure 12).

Using the gender informed indicator from the Monitoring Gender Mainstreaming database yields dramatically different results. Comprehensively, all gender informed HNP projects correspond to 88.8% ($6.2 billion) of the total OECD donor commitments to health sector projects with a gender policy marker from 2013–2015.

## Discussion

### Improved transparency is required to study the Bank’s mainstreaming success in global health

Our analysis of World Bank gender datasets reveals significant discrepancies between its mainstreaming rhetoric and publicly released data on project financing. The World Bank recently claimed that 98% of its total lending (or 97% of its operations) is gender informed. Earlier WID ratings for gender inclusion, which were based on a random sample conducted by the Bank’s Operations and Evaluations Department of 112 Bank projects, also found that 38% of all Bank (and 89% of HNP) projects addressed gender from 1988–1999 ^19^. Such data would appear to show a positive trend in gender considerations for global health and development projects. Indeed, at first glance, data from the Monitoring Gender Mainstreaming database seems largely congruent with this Bank rhetoric. This database shows that over 91% of HNP project lending was ‘gender informed’ in recent years (2013–2016), and that a majority of these projects consider gender at three project dimensions (analysis, actions, and monitoring and evaluation).

However, this information about the gender informed indicator is not included in the PO database, which is the primary resource that external researchers use to identify Bank projects, obtain financial and evaluative information about these projects, and download relevant project documents ^8, 16^. Projects with gender themes within the PO dataset comprised only 1.3% (1985–1989) to 6.2% (2010–2016) of all Bank commitments. Furthermore, there was a concerning lack of congruence between projects included in each project database. The PO and DT databases, which should theoretically contain similar development projects with a gender theme, only had four projects in common, and only one project was listed in all three gender databases. Many projects with a high gender inclusion indicator score (3) did not include a gender theme and were therefore not identifiable in the PO database, but the Bank did not provide any details about how this classification decision was made.

Ultimately, the discrepancies identified through our financial analyses raise a key question: how much can we rely on the datasets that we have analysed? Are critical gender projects missing from the PO and DT databases, and could the trends that we identified in health sector financing simply be inaccurate? The answer is that external researchers have to rely on these databases, because they are the only data publicly released by the Bank on its financing of gender projects. As a study by the Center for Global Development reflected upon in 2016, the Bank’s lack of description of its application of the gender theme (and allocation of its percentage) hampers researchers’ ability to study outcomes and evaluations of gender projects ^19^. The PO and DT databases provide the only way, to the best of our knowledge, to track projects with gender components before 2009, and to track the ways in which gender is included *within* the health sector portfolio, using Bank-assigned health themes.

Even if the gender theme and gender informed indicator are applied consistently across the project databases in the future, analyses of the Bank’s gender portfolio in the health sector will still be limited by the quality of these metrics themselves. The Bank first began scoring projects in its WID portfolio in 1988, using a 0 to 2 rating system (i.e. 0 for no gender inclusion, 1 for gender addressed but no specific actions, and 2 for concrete, specific activities addressing gender or WID issues). However, in 2005, the Bank’s Operations Evaluation Department pointed to a lack of framework for staff accountability and quantitative targets to assess gender projects’ implementation ^20^. Based on its recommendations, the ‘3’ rating was added to the gender indicator, for projects that made recommendations based on gender analysis. In 2010, the Bank’s Independent Evaluation Group underscored the continued absence of a results framework for the indicator ^52^. The gender criteria was adapted, so that gender informed projects were recorded as any with at least a rating of 1, meaning that they take gender into account in *either* the analysis, actions, or monitoring and evaluation dimensions of a project. The rating of each project is determined internally by the Bank, using staff estimates based on a review of project appraisal documents ^19^, and little information is available publicly about the specific criteria for these estimates. Major critiques levelled at the Bank by outside researchers reinforce the Independent Evaluation Group’s finding that this gender informed rating system prioritises gender consideration at the planning project stage ^19, 21^. In particular, based on its analysis of the Monitoring Gender Mainstreaming database and a subset of projects with a gender theme, the Center for Global Development argued that most gender projects do not have gender-specific *outcome* objectives ^19^. The Bank has responded with a new gender strategy (2016–2023), which outlines goals for improved data, staff capacity, results frameworks, and monitoring ^50^. The impact that this new strategy will have on data transparency and gender project classification remains to be seen.

### Health trends from the gender projects databases: framing gender as a women’s issue, targeting middle-income countries, & increasingly turning to ‘innovative financing’

Our analysis of the PO database shows that the Bank invested relatively more than OECD donors in health systems and communicable disease control than reproductive health themes from 1985–2017. This would appear to show a move beyond McNamara and Clausen’s focuses on women’s reproductive roles for development goals. It also falls in line with the Bank’s HNP focuses on universal health coverage and disease control ^53^ However, the PO dataset also indicates that the Bank may have struggled to operationalise its gender as smart economics and mainstreaming frameworks in three ways. These results must obviously be interpreted with caution due to the data transparency issues, and apply to projects with a distinct gender theme.

First, although the 2012 *World Development Report* emphasized including men in gender projects, the Bank and other multilateral health organisations have faced challenges in doing so, as they risk losing their focus on women’s subordination ^5, 54, 55^. Only one project with a gender theme in the PO database had a non-communicable diseases and injuries health theme, and none specifically targeted transgender populations. Yet, the global burden of disease for non-communicable diseases and road injuries is higher for men than women ^9, 52, 56^, the top ten contributors to DALYs (including alcohol and tobacco use) have a greater burden on men than women ^5^, and transgender populations may experience health inequities ^10^. This mirrors a wider problem in global health priority-setting; extremely little emphasis is given to non-communicable diseases, road accidents, and the needs of non-female populations in global public-private partnerships for health (like the Global Fund to Fight AIDS, Tuberculosis and Malaria and the GAVI Alliance) ^14^.

The Bank’s framing of gender as a women’s issue may be related to women’s prominent role in gender staff structure and trainings. According to the most recent evaluation of the Bank’s gender and development policies, 72% of the staff who attended training programmes from 2003–2009 were female and only 13% were from managerial staff levels ^57^. While the Bank’s Independent Evaluation Group was unsure of the actual number of full and part time gender-related staff during this period, they estimated that about 0.8% of staff had a formal gender role ^57^. These figures indicate that, similar to the environment in the 1980s, gender trainings are typically optional and there remains minimal top level staff buy-in for gender projects.

Second, the PO database shows that, for health sector projects with a gender theme, the prime beneficiaries may not be those targeted by the Bank’s wider poverty alleviation agenda. The Gender and Development Board still sits within the Bank’s Poverty Reduction Economic Management (PREM) network ^20^, which should indicate that the majority of its projects target low income populations. It is therefore interesting that the majority of the projects with gender themes in the health sector (PO database) targeted middle income countries, like India, Brazil, and Argentina. This stands in contrast to general OECD donor commitments to the gender marker in the health sector, which primarily targeted Sub-Saharan Africa. This finding has implications for gender equality within the Bank’s poverty alleviation agenda, which is increasingly accepting financing from the private sector.

Third, this embrace of private sector financing for health has made tracking gender projects – and their outcomes – increasingly convoluted. The dip in the Bank’s commitments to all gender projects in the PO and DT databases in 2014 may be due to its investment or channelling of other donors’ investments in trust funds (extra-budgetary or ‘multi-bi’ aid), which allow it to bypass its traditional country-based model. For example, the multimillion dollar Umbrella Fund for Gender Equality (est. 2012) and the new We-Fi facility each have a private window, managed by the IFC ^58^. Similarly, many new innovative financing mechanisms for health at the Bank, like the Global Financing Facility (GFF) ^59^ and the Pandemic Emergency Financing Facility (PEF) ^60^, may have gender components. The new gender trust funds since 2010 may demonstrate the Bank’s move towards financing gender mainstreaming through extra-budgetary and private sources. In a 2017 speech, for instance, President Kim used gender equality as an example of new pathways to bring the private sector into development finance. Associating gender with the private sector and market-based activities could adversely affect the Bank’s gender equality and poverty alleviation goals, so financial sources and channels for gender projects in the health sector should be monitored ^8, 12, 45, 51^.

However, the PO database does not include trust funds, and, while the DT database does include some small gender trust funds in the health sector, it does not include any of these large gender trust funds. Trust funds are not assigned a Bank project number, meaning that it is difficult to systematically obtain project documents and financial data, particularly for closed projects. They are also missing from the Monitoring Gender Mainstreaming database, which only includes projects to which the World Bank contributed directly. This difficulty in tracking gender trust funds is part of a larger issue; researchers have flagged the Bank’s lack of transparency in its use of health sector trust funds and recommended methods to improve data availability ^15^. The Center for Global Development’s analysis using the Monitoring Gender Mainstreaming database demonstrated that, in 2013–2014, the average rating for gender informed projects in the health sector was 2.56, compared to 0.082 in the finance and private sector. This indicates that Bank-financed health projects typically consider gender more than its projects in the private sector. It also underscores that gaging the success of gender mainstreaming in the health sector will require improved trust fund data, including trust fund classification by donor, gender inclusion indicator, and gender theme.

## Conclusion

Most global health organisations and funding agencies have a defined gender policy ^5, 14^. However, institutionalising this policy and developing clear metrics to measure its outcomes have often lacked priority, particularly in the form of financial resources ^21^. The Bill and Melinda Gates Foundation does not currently have metrics in place to measure gender inequalities or women’s empowerment (although its pledge may fill this gap) ^1^, the Global Fund has been criticized for poor monitoring indicators ^14^, and the Institute for Health Metrics and Evaluation (IHME)’s development assistance for health database does not currently have a gender marker. The OECD’s CRS gender equality marker has also been criticised for being ‘based on little knowledge, research or consultation with gender experts’ about the ‘complex ways that men and women are affected differently by development processes’ ^61^.

Ultimately, success in global health comes down to metrics. How institutions define and measure indicators – like gender inclusion markers in Bank projects – determines whether development strategies are meeting their objectives. Indicators for the impact of gender mainstreaming at global health funding agencies should therefore be revisited, particularly in terms of data transparency: why are indicators not universally present, what are they capturing when used, and is this what we need to be monitoring to reach gender equality goals?

In the case of the World Bank, we specifically recommend future research on project outcomes and evaluations for health sector projects with a gender theme. For the time being, this may be best accomplished by selecting all HNP projects in the Monitoring Gender Mainstreaming database from 2013–2016 (i.e. projects with a gender informed indicator of 1–3), locating their project records in the PO database, and looking for gender-specific outcome objectives and results within the Project Appraisal Documents and Project Information Documents. This would update the Center for Global Development’s valuable 2016 study on gender mainstreaming, which suggested that ‘mainstreaming is at best a somewhat paper-based activity at the moment’ ^19^, and would extend its analysis more deeply into the health sector. For a more historical, but less HNP-comprehensive, study, we recommend performing similar document searches for gender-specific indicators and outcome objectives for all health sector projects with a gender theme in the PO database. Such a study would allow for improved understanding of *how* the Bank has operationalised the gender policies outlined in our timeline within the health sector (i.e. whether indicators and their targets have been for women-oriented quotas, gender-specific data disaggregation requirements, gender-equality project outcomes, etc.).

As the World Bank implements its 2016–2023 gender strategy, we recommend two major changes in transparency. First, all past and present Bank projects – including trust funds – should be included in the PO database, and classified by gender theme. The allocation process for this theme should be clearly described. Second, the Bank should revisit the gender informed indicator itself, so that it only includes projects with gender considerations in *all* three dimensions (design, implementation, and evaluation). More information should be released about the criteria used by Bank staff to make these gender inclusion designations for each project and these ratings should be included in the publicly available project databases. The Monitoring Gender Mainstreaming database should either be replaced entirely by a more comprehensive PO database, or should more directly support the Bank’s new gender strategy for 2016–2023, by including more information on gender outcome objectives, and projects before 2009.

Such improved data will foster examination of the impact of gender policies and private investments on poverty reduction and health goals. Ultimately, until gender indicators and revisited and further independent research is conducted, the success of gender mainstreaming in the Bank’s health sector will remain rhetoric rather than reality.

## Data availability

Data for this article are available on OSF: http://doi.org/10.17605/OSF.IO/FDHGM ^62^

Data are available under the terms of the Creative Commons Zero "No rights reserved" data waiver (CC0 1.0 Public domain dedication).
