# Supplementary material for: Mainstreaming as rhetoric or reality? Gender and global health at the World Bank
Source: Wellcome Open Res. 2018 Aug 17;3:18. Originally published 2018 Feb 26. [Version 2] doi: 10.12688/wellcomeopenres.13904.2 (PMC6113881; doi:10.12688/wellcomeopenres.13904.2)
Supplement: Peer review history

# Peer review: Referee response for version 2

**DOI:** 10.21956/wellcomeopenres.16069.r33708

**Published:** 2018-08-28

**Authors:** Krishna Hort

**Affiliations:** 1 Nossal Institute for Global Health, University of Melbourne, Melbourne, Australia

Thanks for opportunity to review the revised version of this paper.

 Overall, the authors are to be congratulated as the revised paper has addressed the issues raised in my review of the initial version, and is considerably clearer in structure and in the description of methodology. I also note that the authors have taken the opportunity to undertake some additional analysis, which has further strengthened the paper.

 An important change from the original version is the increased focus on the issues of transparency and the accuracy and reliability of the data provided publicly by the World Bank. The additional analysis using the Monitoring Gender Mainstreaming database, and search for duplicates across the different databases, identified further discrepancies and inconsistencies in the data, and the conclusions and recommendations have an increased focus on this aspect.

 As a further comment, the paper now provides an interesting example of a methodology to measure transparency and reliability in reporting from project databases, through a comparison of consistency of identification across different databases. This resembles the ‘capture – recapture’ methodology used by epidemiologists to measure the coverage of surveillance systems. It is perhaps something that could be adapted to measure other aspects of information captured in multiple databases.

 I have no further significant concerns with paper.

 Responses to recommendations:

1. Inconsistencies in Purpose / objectives – resolved. The revised papers refers to scoping review in the introduction and identifies the purpose as a roadmap for future research. This is a much clearer explanation of purpose and contribution.
2. Clarifying gender theme / gender informed – addressed. The revisions in the methods section explains the classification of gender projects. Table 1 provides useful definition of terms.
3. Figure 2 comparing finance in total / health sector / gender theme is a useful addition and addresses concerns re: interpretation table 3.
4. Governance of gender replaced with conceptualisation of gender – resolved.
5. Absence of methodological framework – resolved. Such a framework would be needed for a case study, but the current content is adequate for the revised purpose and objective. The shift in focus from policy implementation to focus on indicators and publicly available data – appropriate.
6. Inclusion / exclusion criteria – addressed.
7. Order of analysis and check for duplicates – addressed. Addition of Monitoring Gender Mainstreaming (MGM) database- gender indicator scores and comparison with PO and DT databases – valuable additions that strengthen the paper. The comparison of gender theme / gender informed projects is very illuminating.
8. Results – figure 2 addresses the link between financing and phases. The identification of only 4 common projects between PO and DT databases is very illuminating and provides further evidence of the lack of consistency in the application of project definitions.
9. Discussion & conclusions – is now better structured, and addresses the potential limitations up front. There is much more acknowledgement of the issues of transparency and inconsistencies in data sources in responding to WB claims on gender financing.  Lack of congruence emerges as key issue, not previously raised.
10. New discussion on gender theme and indicator addresses the concerns about measurement of mainstreaming using these project definitions, and the greater sensitivity of gender measurement of projects does respond to this question. Much more focused conclusions and recommendations for further study.

I have read this submission. I believe that I have an appropriate level of expertise to confirm that it is of an acceptable scientific standard.

# Peer review: Referee response for version 2

**DOI:** 10.21956/wellcomeopenres.16069.r33709

**Published:** 2018-08-20

**Authors:** Gavin Yamey, Kaci Kennedy

**Affiliations:** 1 Center for Policy Impact in Global Health, Duke Global Health Institute, Duke University, Durham, NC, USA

Many thanks for the opportunity to review the revised version of this important study. We very much appreciate the revisions, including the clarification of this study as a scoping review, the additional analysis of the Monitoring Gender Mainstreaming database, the expanded discussion of challenges with data availability, and the excellent new Figure 2. The authors have greatly strengthened the paper, which is an important addition to the literature.

We have read this submission. We believe that we have an appropriate level of expertise to confirm that it is of an acceptable scientific standard.

# Peer review: Referee response for version 1

**DOI:** 10.21956/wellcomeopenres.15116.r32503

**Published:** 2018-04-09

**Authors:** Krishna Hort

**Affiliations:** 1 Nossal Institute for Global Health, University of Melbourne, Melbourne, Australia

Thanks for the opportunity to review and comment on this paper. The paper is addressing an important question, and, by providing an external analysis of World Bank data, is contributing to independent scrutiny of development policy and funding. While the paper is only partly successful in its aim of comparing World Bank gender policies with financing, it does draw attention to issues in the availability and usability of World Bank data that are deserving of attention by the Bank and other development agencies.

1. There are inconsistencies in the presentation, particularly in terms of purpose and objectives.
  (1) Inconsistencies in how the study is described – its purpose and objectives. The abstract refers to ‘ a case study of how global health organisations frame their gender policies and measure their success’. While page 3 column 2 refers to a comparison of ‘this policy.. with financing for gender in the health sector’. The paper describes the evolution of gender policies at the WB and seeks to interpret this in terms of framing; but much of the emphasis is on measurement of the proportion of financing for gender projects. The issue of measurement is raised, but in terms of the transparency of databases, rather than how success is measured.
  (2) The paper compares the proportion of Bank funding on gender projects with the proportion of projects that are ‘gender informed’ in three places (abstract, page 3 and page 15).  There is an inconsistency in the percentage quoted as gender informed ( 98% in the abstract and discussion (page 15), and 97% on the last line column 1 of page 3). But more significantly, this comparison itself seems misleading, as one assumes that there are projects that are not ‘gender projects’ but that are informed by gender. The lack of precision on definition of the terms ‘gender informed’ and ‘gender projects’  (as raised in the discussion) raises further questions about the comparison.
  (3) The references in the text to Tables 2 and 3 appear to be incorrect on page 8 column 1, or the tables are incorrectly labelled.  The percentage of WB funding in the health sector is provided in Table 3 (not Table 2); while the definition of themes is provided in Table 2 (not Table 3).
2. Study design and methods
  In terms of methods, the study is described as a ‘case study’.  However,  a conceptual framework for the study, and in particular the conceptual basis for the proposed comparison between policy and programs, is not presented.
  There is an assumption that policy should be reflected in financing.  But, noting that four phases of policy are described over several time periods, it is not clear which policy is being compared to which funding, over what time period.
  The expected linkage between policy statements and project financing is not described.  Here more explanation of the process by which policy statements are ‘translated’ into project financing would assist in understanding what might be expected in terms of project financing and how it might reflect policy changes.
  Some key terms are not defined  - for example what is meant by ‘governance of gender’ ? (it appears to refer mainly to a description of policy formulation, rather than governance in the sense of institutional roles and relationships).
3. Details of methods and analysis
  Some details of the methods and analysis are also missing.
  The criteria for inclusion / exclusion of the events and policies listed in the Timeline of Figure 1 is not provided. The process of selection of the events and policies for the timeline could be clarified.
  The process for construction of Figure 2 could also be further clarified. It appears that the initial step in analysis of both databases was to identify projects with gender themes, and then to exclude non-health projects.  Would it have made a difference if the process was reversed, and health projects selected first, then non-gender removed ?  Was there a rationale for the start with gender projects ?  It is also not clear whether there is a potential for projects to be included in both databases, and whether a check for duplicates was undertaken. The labelling for Figure 2 should note that the dollar amounts are in USD million.
4. Results
  It is difficult to interpret the graphs and figures on funding by theme and period without some information on the overall funding envelope in health and gender over the period. This is provided in Table 3, but there is little text description of the information in Table 3, except for Page 8 para 2 (incorrectly referred to as Table 2); and in the discussion, page 15 (where figures are provided from Table 3, but without the reference to the table). There does not appear to be any effort to relate the changes in project financing with the policy phases described in Figure 1.
5. Discussion and conclusions
  The key finding from the paper is a disjunction claimed between policies on gender mainstreaming and the proportion of funds allocated to gender projects. This is contrasted with the Bank’s claim on the proportion of ‘gender informed’ lending.
  The discussion includes an examination of the limitations encountered in obtaining and interpreting the data.  These limitations suggest, however, more caution in some of the authors’ claims, given the questions on the completeness and definitions used in the project databases, and lack of clarity on the measurement of ‘gender informed’.
  More fundamentally, the issue of the measurement of ‘gender informed’ projects raises an inconsistency which lies at the heart of what the study aims to do. Is it possible to measure the success of a gender mainstreaming policy by the proportion of funds allocated to ‘gender projects’? Surely the success of gender mainstreaming would be seen in a decrease in gender specific projects, and an increase in the incorporation of gender into all other projects.  The paper addresses the issue of the meaning of ‘gender informed’, but has not questioned the fundamental assumption of whether measurement of allocation of funding to ‘gender projects’ is an appropriate way to measure the implementation of gender policies, particularly if the policy direction is towards ‘mainstreaming’.
  Perhaps the most important finding from the study is the difficulty faced by independent observers in assessing and measuring the extent to which a key global organisation translates its policies into program actions. The proportion of funding allocated to projects satisfying specific gender criteria may be a crude measure of the application of gender inclusion, but, in the absence of other data, they become the default measure. As the authors conclude, how we measure our achievements determines whether they will be classed as successful or not. Their recommendations for improved reporting and a clearer ‘gender informed project’ indicator would go some way to address the deficiencies found.

I have read this submission. I believe that I have an appropriate level of expertise to confirm that it is of an acceptable scientific standard, however I have significant reservations, as outlined above.

# Response

**Published:** 2018-08-10

**Authors:** Janelle Winters

**Affiliations:** University of Edinburgh, UK

***We have submitted a new version (version 2) of our article, which should be released in August 2018. Our responses to Dr. Krishna Hort's valuable feedback are below (in bold).***

 Thanks for the opportunity to review and comment on this paper. The paper is addressing an important question, and, by providing an external analysis of World Bank data, is contributing to independent scrutiny of development policy and funding. While the paper is only partly successful in its aim of comparing World Bank gender policies with financing, it does draw attention to issues in the availability and usability of World Bank data that are deserving of attention by the Bank and other development agencies.

**Thank you for your detailed feedback. We agree that the paper could benefit from tightening our aim. In the abstract and introduction, we have clarified that our research study is intended as a scoping review of general gender policy development at the Bank and of how projects with gender themes at the Bank have been financed in the health sector, rather than a case study of gender policy at the Bank. To our knowledge, this is the first external analysis to produce a timeline of gender policies at the Bank, and to explore gender project financing in the health sector. We hope that future research will be able to use this paper as a base to compare gender policies and financing for gender-incorporating projects in global health in more depth, such as through case studies. As you rightly point-out, one of our take-home points is that comparing policy and financing for gender projects, particularly in specific sectors like health, is hampered by publicly available data. Ideally we would have been able to track financing for all health projects with a gender theme across all of our phases (1972-2017), but we were limited by data availability. We reinforce the data availability angle in our conclusion, through an adapted final paragraph on transparency, and in our discussion, through a more concrete discussion of how the gender informed indicator evolved at the Bank and the limitations of each database in capturing this indicator.**

 There are inconsistencies in the presentation, particularly in terms of purpose and objectives.

 (1) Inconsistencies in how the study is described – its purpose and objectives. The abstract refers to ‘ a case study of how global health organisations frame their gender policies and measure their success’. While page 3 column 2 refers to a comparison of ‘this policy.. with financing for gender in the health sector’. The paper describes the evolution of gender policies at the WB and seeks to interpret this in terms of framing; but much of the emphasis is on measurement of the proportion of financing for gender projects. The issue of measurement is raised, but in terms of the transparency of databases, rather than how success is measured.

**You are correct to point-out that our use of the term ‘case study’ is confusing. We have refined the abstract to indicate that we are summarizing the development of the Bank’s gender conceptualization and framework, and analysing its financing for gender projects in the health sector since 1985, rather than a case study.**

**We also realise that we may have introduced confusion by not clearly defining the different ways that the Bank defines (measures) its gender projects. We have updated our methodology and our use of the term ‘gender project’ throughout the manuscript to address this issue. In short, the project databases that we use (Development Topics [DT] and Projects & Operations [PO]), the gender ‘theme’ (as described in Table 1) is what we used to classify a project as a ‘gender project’. This theme was assigned by Bank staff, and little information is available about how projects are classified by theme and why the datasets would include so many different projects for each theme. This theme classification does not mean that a project was *primarily *a gender project. Instead, it means that the project was assigned a gender theme percentage (between 5-100%), among up to four other themes (such as health system strengthening, etc., as shown in Tables 1 and 2). We considered any project with any gender theme percentage a ‘gender project’, although very few were exclusively gender focused. Additionally, the Bank has used a ‘gender informed’ indicator to report the percentage of its overall operations and the percentage of its financing that are gender informed (this is described in our discussion). Again, Bank staff assign this indicator, and it is given to a project if it considers gender at *any *of three levels of the project cycle. Unfortunately, it is not possible to search the DT and PO using the gender informed indicator (which is an issue, as we point-out in the revised discussion). We now suggest in the conclusion specific ways that the Bank could improve its transparency, using the gender theme and indicator, and productive future research for external researchers.**

 (2) The paper compares the proportion of Bank funding on gender projects with the proportion of projects that are ‘gender informed’ in three places (abstract, page 3 and page 15).  There is an inconsistency in the percentage quoted as gender informed ( 98% in the abstract and discussion (page 15), and 97% on the last line column 1 of page 3). But more significantly, this comparison itself seems misleading, as one assumes that there are projects that are not ‘gender projects’ but that are informed by gender. The lack of precision on definition of the terms ‘gender informed’ and ‘gender projects’  (as raised in the discussion) raises further questions about the comparison.

**Actually, there is not a consistency in our reporting of the percentage of gender informed projects, although we understand why this would appear to be the case. The Bank reported the percentage of FY13 Bank projects that were gender informed in two ways: as a percentage of all operations (98%, based on the number of projects), and as a percentage of total funding (97%, based on the aggregate commitments to these projects). We have inserted parentheses to make this distinction more evident, and clearly cited each figure.**

****

**We also see why it would seem unfair to compare ‘gender informed’ and ‘gender projects’, particularly if gender projects had a primary gender focus and gender informed projects merely had to have some sort of smaller-scale gender consideration. Based on our above description of the gender theme and gender informed indicator, we recommend that the Bank be more clear in its application of and inclusion/exclusion criteria for the ‘gender theme’ to projects, especially because some projects only have a small percentage for their gender theme (5-10%). When we wrote our first draft of the paper, we were unaware that the World Bank had a publicly searchable database, through its World Bank Finances portal (rather than its Projects & Operations or Development Topics project portals). This Monitoring Gender Mainstreaming database includes a list of projects, only some of which are classified by sector, with their gender indicator score and sometimes whether they have a gender theme. We have analysed the gender indicator scores and presence of a gender theme for each project in the HNP sector, and included this analysis in our methodology, results, and discussion. We find that the gender informed indicator is poorly correlated with a gender theme, and that it is unclear how designations of indicator and theme are made internally at the Bank.**

****

**As we now suggest in the conclusion, the Bank should make its gender informed indicator searchable in the PO database, so that scholars could use this indicator instead of project themes to track gender financing. They should also release more historic data, since the Monitoring Gender Mainstreaming database only includes partial data for the years 2009-2016. Based on your comment, we have replaced the term ‘gender projects’ throughout the manuscript with the phrase ‘projects with a gender theme’, and have more deliberately defined the difference between themes and informed indicators. We hope that this makes our comparisons between the gender informed indicator, gender theme, and wider dialogue about mainstreaming clearer.**

 (3) The references in the text to Tables 2 and 3 appear to be incorrect on page 8 column 1, or the tables are incorrectly labelled.  The percentage of WB funding in the health sector is provided in Table 3 (not Table 2); while the definition of themes is provided in Table 2 (not Table 3).

**Thank you for catching this mistake – this was our error. We have corrected it, and also added a graph (Figure 2, based on Table 3 data) to more visually compare projects with a gender theme to the phases in our timeline.**

 Study design and methods

 In terms of methods, the study is described as a ‘case study’.  However,  a conceptual framework for the study, and in particular the conceptual basis for the proposed comparison between policy and programs, is not presented.

 There is an assumption that policy should be reflected in financing.  But, noting that four phases of policy are described over several time periods, it is not clear which policy is being compared to which funding, over what time period.

 The expected linkage between policy statements and project financing is not described.  Here more explanation of the process by which policy statements are ‘translated’ into project financing would assist in understanding what might be expected in terms of project financing and how it might reflect policy changes.

 Some key terms are not defined  - for example what is meant by ‘governance of gender’ ? (it appears to refer mainly to a description of policy formulation, rather than governance in the sense of institutional roles and relationships).

**As addressed above, we have removed the word ‘case study’ to avoid confusion. We have also removed the word ‘governance’, because you are correct that we are not considering policy through the lens of institutional structures or decision-making processes. We have replaced it with ‘conceptualisation’ of gender, which more clearly captures our interest in how the Bank’s framing of gender has evolved over time and been generally operationalised.**

****

**We see value in your point that we could describe the link between policy statements and project financing (i.e. provide a methodological framework) in more depth. However, tracking financing as a proxy for understanding institutional priorities is widely accepted in global governance (see, for instance, J Shiffman, ‘Donor funding priorities for communicable disease control in the Developing World’, *Health Policy & Planning *2006, 21:6, pp. 411-420; D Sridhar et al., ‘World Bank’s financing, priorities, and lending’, *BMJ *2017, 358; L Ferguson and S Harman, ‘Gender and infrastructure in the World Bank’, *Development Policy Review *2015 33:5, pp. 653-671). We therefore feel that it is beyond the scope of the paper to delve into methodological frameworks, particularly because our emphasis is more on transparency issues with indicators. We hope that readers will see our two results sections – the gender conceptualization timeline and finance tracking – as largely independent analyses, and have therefore scaled-down our discussion of rhetoric and reality in the context of policy implementation (and scaled-up our discussion of rhetoric and reality in the context of indicators and publicly available data) in both the introduction and discussion.**

 Details of methods and analysis

 Some details of the methods and analysis are also missing.

 The criteria for inclusion / exclusion of the events and policies listed in the Timeline of Figure 1 is not provided. The process of selection of the events and policies for the timeline could be clarified.

**We have added a sentence in the methodology to more explicitly explain our inclusion/exclusion criteria for our events and policies timeline.**

 The process for construction of Figure 2 could also be further clarified. It appears that the initial step in analysis of both databases was to identify projects with gender themes, and then to exclude non-health projects.  Would it have made a difference if the process was reversed, and health projects selected first, then non-gender removed ?  Was there a rationale for the start with gender projects ?  It is also not clear whether there is a potential for projects to be included in both databases, and whether a check for duplicates was undertaken. The labelling for Figure 2 should note that the dollar amounts are in USD million.

**Thank you for noting that we are missing units in (what was then) Figure 2; we have corrected this omission (see Figure 3). We are grateful for your useful suggestion that we check for duplicates in both datasets; we were surprised to find that only four projects overlapped between the two project databases (i.e. very few projects were included in both datasets) and that only one project overlapped between all three databases. This reinforces the poor consistency of the Bank’s use of the gender theme. Because gender theme percentages are assigned to all projects that we included in the PO dataset (and all projects in the DT dataset had a gender theme), theoretically the number of projects with a gender theme should be identical in both datasets. We have added a sentence in the methodology about checking for duplicates, and raised the issue in the results.**

****

**It does not make a difference to reverse the analysis steps, by sorting first for gender themes and then excluding health projects in the PO database. Each project is tagged in the databases by sector (i.e. health) and theme (i.e. gender, and other health themes) in the PO database. In the DT database, the Bank has already sorted projects into a gender category, so it is not possible to perform a reverse analysis. Future research could, instead of using pre-assigned themes, go through each project document from the PO dataset manually, and search for the word gender; this could shed light on the accuracy of the theme designation. As we suggest in the revised conclusion, future research could also attempt to bridge the Monitoring Gender Mainstreaming (which does not include project documents) and PO database (which includes project documents) for recent years, to track the association of the gender theme and gender inclusion indicator with gender-specific outcomes measures. This would go a long way to bridging the gap in policy dialogue with financing and project-level data, but would take months of data analysis and goes beyond the scope of our study.**

 Results

 It is difficult to interpret the graphs and figures on funding by theme and period without some information on the overall funding envelope in health and gender over the period. This is provided in Table 3, but there is little text description of the information in Table 3, except for Page 8 para 2 (incorrectly referred to as Table 2); and in the discussion, page 15 (where figures are provided from Table 3, but without the reference to the table). There does not appear to be any effort to relate the changes in project financing with the policy phases described in Figure 1.

**Thank you for raising this point, which was also highlighted by our other reviewer. We have added a graph (Figure 2), with more description, which combines data from Table 3 across three of our timeline’s phases (phases II-IV). We would like to be able to compare project financing with gender themes before 1985, but no data has been released for this earlier phase. As much as we would have also liked to be able to focus explicitly on gender conceptualisation and policy for health in our timeline section (which would have facilitated direct comparison with our gender and health project financing analysis), we found extremely little literature on gender and health policy at the World Bank. We hope that this paper, and potentially improved transparency at the Bank, provides background for further research on gender policy and health.**

 Discussion and conclusions

 The key finding from the paper is a disjunction claimed between policies on gender mainstreaming and the proportion of funds allocated to gender projects. This is contrasted with the Bank’s claim on the proportion of ‘gender informed’ lending.

 The discussion includes an examination of the limitations encountered in obtaining and interpreting the data.  These limitations suggest, however, more caution in some of the authors’ claims, given the questions on the completeness and definitions used in the project databases, and lack of clarity on the measurement of ‘gender informed’.

**You are correct that it is important to use caution in our claim. We had a paragraph in the discussion reinforcing this fact; that one could argue that all of our data analysis on projects with a gender theme does not capture the Bank’s true gender inclusion in global health, but that this limitation merely highlights the poor data available to external researchers for understanding the Bank’s gender portfolio. We have moved this paragraph earlier into the discussion, and qualified some of our findings from our financing analysis. As described earlier in our response, we have added recommendations to address these limitations in the discussion and conclusion.**

 More fundamentally, the issue of the measurement of ‘gender informed’ projects raises an inconsistency which lies at the heart of what the study aims to do. Is it possible to measure the success of a gender mainstreaming policy by the proportion of funds allocated to ‘gender projects’? Surely the success of gender mainstreaming would be seen in a decrease in gender specific projects, and an increase in the incorporation of gender into all other projects.  The paper addresses the issue of the meaning of ‘gender informed’, but has not questioned the fundamental assumption of whether measurement of allocation of funding to ‘gender projects’ is an appropriate way to measure the implementation of gender policies, particularly if the policy direction is towards ‘mainstreaming’.

**We believe that our above discussion of the gender theme and gender indicator respond to this concern that tracking ‘gender projects’ is not a fair way to measure implementation of mainstreaming.**

 Perhaps the most important finding from the study is the difficulty faced by independent observers in assessing and measuring the extent to which a key global organisation translates its policies into program actions. The proportion of funding allocated to projects satisfying specific gender criteria may be a crude measure of the application of gender inclusion, but, in the absence of other data, they become the default measure. As the authors conclude, how we measure our achievements determines whether they will be classed as successful or not. Their recommendations for improved reporting and a clearer ‘gender informed project’ indicator would go some way to address the deficiencies found.

**We agree that data availability and transparency in use of indicators may be our most important take-home messages. Thank you again for your detailed feedback.**

# Peer review: Referee response for version 1

**DOI:** 10.21956/wellcomeopenres.15116.r31183

**Published:** 2018-03-26

**Authors:** Gavin Yamey, Kaci Kennedy

**Affiliations:** 1 Center for Policy Impact in Global Health, Duke Global Health Institute, Duke University, Durham, NC, USA

Thank you for giving us the opportunity to peer review this interesting and potentially important study. 

 Janelle Winters and colleagues have conducted an analysis of the World Bank’s gender policies and the Bank’s financing of gender programs.  As discussed further below, there have been several previous analyses of the World Bank’s gender projects, but Winters and colleagues’ study applies a specific global health lens, which appears to be novel. 

 There are two parts to the study, which are somewhat disconnected. The first part is a literature review, reviewing both the peer-reviewed and grey literature, to construct a “timeline” of the evolution of the Bank’s gender focus. The second part is a quantitative financial analysis of the Bank’s spending on gender projects. The timeline resulting from part 1 is shown as a figure (Figure 1) and also described through 4 key phases (women in development [WID]; institutionalization of WID; gender mainstreaming; gender equality through ‘smart economics’). This timeline is very valuable.  We think it will provide a helpful “roadmap” for others who wish to conduct research related to the World Bank’s gender portfolio in the future.  The results of Part 2 suggest that from 1985-2016, the Bank committed only between 1.2-6.2% of IBRD and IDA commitments to “gender projects.”

 Below we comment on the importance, originality, validity, presentation, and interpretation of the research.

Importance of research question

 The role of gender in development has gained increasing momentum and has moved higher up the agenda with SDG 6, “achieve gender equality and empower all women and girls.”

 Many development and health agencies, including the World Bank, make claims about how they are mainstreaming gender in their work.  An external assessment such as this one is an important way to keep the World Bank accountable and provides a foundation to advocate for changes if results deem it necessary.

 This study is also very timely indeed given that the Swedish Institute for Global Health Transformation has just launched a new Lancet Commission exploring the links between SDGs 3 (health), 5 (women and girls), and 16 (institutions) (one of us, GY, is a Commissioner). Understanding how a major development institution approaches gender is helpful for that Commission’s work.

Originality of the research

 We would like to make two points on originality. First, one of us (GY) used to be a journal editor, at the BMJ and PLOS, and at both publishers we would not let authors claim that there has “never” been a similar study.  What we asked authors to say, out of caution, is something like “to the best of our knowledge, there have been no previous studies that examined X and we believe ours is the first.”  This terminology allows for the possibility that you may have missed a study (e.g. in a non-English language, in the grey literature, in a consulting report, by the Bank itself, etc.).

 Second, there have been several studies of the World Bank’s gender policies in recent years and we think it would be helpful for the authors to more explicitly review and summarize what these found. Perhaps the most high profile was Kenny and O’Donnell’s study “Do the Results Match the Rhetoric? An Examination of World Bank Gender Projects,” published by CGD. That study used a Bank dataset, “Monitoring Gender Mainstreaming in World Bank Lending Operations,” for its analysis (with 1666 projects that date from July 2009 to June 2014).  We also note that the Bank itself conducted a study of its gender policy that included health sector projects (Evaluating a Decade of World Bank Gender Policy: 1990–99, World Bank Operations Evaluation Department, 2005).

Validity of the research

 Overall, the methods seem appropriate and the mix of a more qualitative literature review with a quantitative financial analysis is a strength. However, as mentioned, it would be helpful to better connect these two distinct parts of the study (one easy way to do this, for example, would be to display key measurements/commitments in each phase of World Bank gender policies alongside financial data from the years during that phase).  It was excellent, and highly valuable, to see a comparison of the World Bank’s financial data from the PO and DT databases with a broader analysis of development assistance for gender projects using the CRS database. The breakdown of gender funding in the health sector by theme and also by recipient country are also interesting and helpful.

 Below, we make a few specific comments about the overall approach and we note some minor inconsistencies or possible errors.

 First, it is heartening to see in the introduction a discussion of men and the LGBTI community. As the authors note, in many parts of the world, men’s health outcomes are much worse (e.g. the IHME’s GBD2010 study found that women in the Russian Federation were outliving men by an average of 11.6 years).  In a paper that one of us co-authored (reference 9 in Winters *et al*’s paper), we note that, “In many societies, men generally enjoy more opportunities, privileges and power than women, yet these multiple advantages do not translate into better health outcomes.” This is likely to be due to a combination of factors, including risk-taking behavior, occupational exposure to risk factors, gendered norms of male behavior, etc. While the introduction makes this point, it is not clear whether the authors specifically examined whether the World Bank has any specific policies on or funding for men’s health or LGBTI health. EMRO (WHO Europe) is developing its first men’s health strategy (due to be published in September 2018) and PAHO is also working on this issue, so it would be good to know where the Bank is.

 Second, while it is clearly highly appropriate to use the gender policy marker for analyzing projects in the CRS database, it would be helpful to know if there are any data on how well this marker captures gender-specific project financing.  Might some projects be missed?  Would there be value, for example, in taking a sample of projects that were not captured by the marker to see if gender was included?

 Third, in Figure 2, the numbers from the Development Topics Database don’t seem right—it starts with n = 90, then 8 were dropped, but then the figure still says 90 (after dropping 8, it should say 82). In addition, we think it would be good to mention the date restriction in figure 2 itself and not just in the methods section. Looking at figure 2 right now, readers may think the search was from January 1, 1985 through December 31, 2017 (the figure says 1985-2017, and this would give 92 projects); the methods section states a narrower date range, i.e. to July 1, 2017 (which yields 90 projects).

 Fourth, it would be good to update the projects, since the World Bank now lists 102 gender projects: http://www.worldbank.org/en/topic/gender/projects/all.

Presentation

 The presentation is generally clear.

Interpretation

 The conclusions focus primarily on the shortcomings of the reporting mechanisms and indicators for gender mainstreaming in general.  Gender reporting shortcomings were also a key theme in the CGD study on World Bank gender rhetoric.  It would be valuable, we think, to discuss the concrete concerns with the gender project evaluation criteria moving forward and the conclusions could align more with the specific research question on gender-focused health programs.

 There is one comparison that the authors make that we think may not be a valid one. The authors note “the Bank’s recent claim that 98% of its total lending is gender informed” and then they compare this 98% figure with the proportion of IBRD/IDA financing that is specifically for gender projects.  Is this really an apples to apples comparison?  Being “gender informed” is not the same as financing a gender project.

We have read this submission. We believe that we have an appropriate level of expertise to confirm that it is of an acceptable scientific standard, however we have significant reservations, as outlined above.

# Response

**Published:** 2018-08-10

**Authors:** Janelle Winters

**Affiliations:** University of Edinburgh, UK

***We have submitted a new version (version 2) of our article, which should be released in August 2018. Our responses to Dr. Gavin Yamey and Dr. Kaci Kennedy's feedback are below (in bold).***

 Thank you for giving us the opportunity to peer review this interesting and potentially important study. 

**Thank you for all of your valuable comments; they are very appreciated, and we have done our best to refine our analysis to incorporate them.**

 Janelle Winters and colleagues have conducted an analysis of the World Bank’s gender policies and the Bank’s financing of gender programs.  As discussed further below, there have been several previous analyses of the World Bank’s gender projects, but Winters and colleagues’ study applies a specific global health lens, which appears to be novel. 

 There are two parts to the study, which are somewhat disconnected.

 The first part is a literature review, reviewing both the peer-reviewed and grey literature, to construct a “timeline” of the evolution of the Bank’s gender focus. The second part is a quantitative financial analysis of the Bank’s spending on gender projects. The timeline resulting from part 1 is shown as a figure (Figure 1) and also described through 4 key phases (women in development [WID]; institutionalization of WID; gender mainstreaming; gender equality through ‘smart economics’). This timeline is very valuable.  We think it will provide a helpful “roadmap” for others who wish to conduct research related to the World Bank’s gender portfolio in the future.  The results of Part 2 suggest that from 1985-2016, the Bank committed only between 1.2-6.2% of IBRD and IDA commitments to “gender projects.”

**We appreciate this feedback, and also felt that it was challenging to insert global health frameworks and policies explicitly into our ‘roadmap’ for gender at the Bank (although we like this phrase and have borrowed it in the manuscript). In the abstract and introduction, we have clarified that our research study is intended as a scoping review of general gender policy development at the Bank and of how projects with gender themes at the Bank have been financed in the health sector, rather than a case study of gender policy at the Bank. We hope that the literature review provides a strong context for readers to understand and discuss the financial results.**

**Specifically, we intend this paper to act as a base for future research to compare gender policies and financing for gender-incorporating projects in global health in more depth, as suggested in the revised conclusion. Ideally we would have been able to track financing for all health projects with a gender theme across all of our phases (1972-2017), but we were limited by data availability. We reinforce the data availability angle in our conclusion, through an adapted paragraph on transparency, and in our discussion, through a more concrete discussion of how the gender informed indicator evolved at the Bank and the limitations of each database in capturing this indicator.**

 Below we comment on the importance, originality, validity, presentation, and interpretation of the research.

Importance of research question

 The role of gender in development has gained increasing momentum and has moved higher up the agenda with SDG 6, “achieve gender equality and empower all women and girls.”

 Many development and health agencies, including the World Bank, make claims about how they are mainstreaming gender in their work.  An external assessment such as this one is an important way to keep the World Bank accountable and provides a foundation to advocate for changes if results deem it necessary.

 This study is also very timely indeed given that the Swedish Institute for Global Health Transformation has just launched a new Lancet Commission exploring the links between SDGs 3 (health), 5 (women and girls), and 16 (institutions) (one of us, GY, is a Commissioner). Understanding how a major development institution approaches gender is helpful for that Commission’s work.

**Thank you for this point – we agree that considering how gender is incorporated in health sector projects is particularly timely, and encourage future research using the three publicly available databases that we describe.**

Originality of the research

 We would like to make two points on originality. First, one of us (GY) used to be a journal editor, at the BMJ and PLOS, and at both publishers we would not let authors claim that there has “never” been a similar study.  What we asked authors to say, out of caution, is something like “to the best of our knowledge, there have been no previous studies that examined X and we believe ours is the first.”  This terminology allows for the possibility that you may have missed a study (e.g. in a non-English language, in the grey literature, in a consulting report, by the Bank itself, etc.).

**We agree and have revised our claims throughout the paper. We very much appreciate you raising this constructive point.**

 Second, there have been several studies of the World Bank’s gender policies in recent years and we think it would be helpful for the authors to more explicitly review and summarize what these found. Perhaps the most high profile was Kenny and O’Donnell’s study “Do the Results Match the Rhetoric? An Examination of World Bank Gender Projects,” published by CGD. That study used a Bank dataset, “Monitoring Gender Mainstreaming in World Bank Lending Operations,” for its analysis (with 1666 projects that date from July 2009 to June 2014).  We also note that the Bank itself conducted a study of its gender policy that included health sector projects (Evaluating a Decade of World Bank Gender Policy: 1990–99, World Bank Operations Evaluation Department, 2005).

**We had referenced both papers, but you are correct that we did not engage with them optimally. Kenny and O’Donnell’s (Center for Global Development, CGD) study provides a base for many of our points raised in the discussion about the Bank’s gender and health financial portfolio (which we have now restructured, to highlight them more explicitly). Upon taking a fresh look at this study, we realised that this database on gender and global health, Monitoring Gender Mainstream is available through the Bank’s Finances portal. It has also been updated to include two years of projects since the CGD study (now 2060 projects). We therefore performed a basic analysis of this third database for all projects with a HNP global practice designation. This database is especially helpful for comparing overlaps between the gender informed indicator and the gender theme, but is not searchable by health theme and only includes HNP data from 2013-2016. Details of this analysis are in the methods and results.**

**We have added a paragraph in the discussion about the gender inclusion indicator, and how it evolved at the Bank. This draws on both the 2016 CGD study and the Operations Evaluations department (OED) 2005 study.**

Validity of the research

 Overall, the methods seem appropriate and the mix of a more qualitative literature review with a quantitative financial analysis is a strength. However, as mentioned, it would be helpful to better connect these two distinct parts of the study (one easy way to do this, for example, would be to display key measurements/commitments in each phase of World Bank gender policies alongside financial data from the years during that phase).  It was excellent, and highly valuable, to see a comparison of the World Bank’s financial data from the PO and DT databases with a broader analysis of development assistance for gender projects using the CRS database. The breakdown of gender funding in the health sector by theme and also by recipient country are also interesting and helpful.

**This is another helpful suggestion. We have added Figure 2, which compares Bank health sector commitments and commitments to projects with a gender theme during the final three phases (1985-2016, PO database). Data is not available to track health commitments for projects with a gender theme or gender inclusion indicator before these dates.**

 Below, we make a few specific comments about the overall approach and we note some minor inconsistencies or possible errors.

 First, it is heartening to see in the introduction a discussion of men and the LGBTI community. As the authors note, in many parts of the world, men’s health outcomes are much worse (e.g. the IHME’s GBD2010 study found that women in the Russian Federation were outliving men by an average of 11.6 years).  In a paper that one of us co-authored (reference 9 in Winters *et al*’s paper), we note that, “In many societies, men generally enjoy more opportunities, privileges and power than women, yet these multiple advantages do not translate into better health outcomes.” This is likely to be due to a combination of factors, including risk-taking behavior, occupational exposure to risk factors, gendered norms of male behavior, etc. While the introduction makes this point, it is not clear whether the authors specifically examined whether the World Bank has any specific policies on or funding for men’s health or LGBTI health. EMRO (WHO Europe) is developing its first men’s health strategy (due to be published in September 2018) and PAHO is also working on this issue, so it would be good to know where the Bank is.

**This is certainly an interesting comment, and we agree that it would be helpful to know where the Bank stands on issues related to men and the LGBTI community. However, we feel that this goes beyond the scope of our paper; we did not encounter specific Bank policies in the published papers that we reviewed (although we did not complete a systematic review and performed the review last August, so it is possible that we missed some references) and did not find health themes related to LGBTI or men’s health issues in the PO database. We would certainly recommend that future research looks into the Bank’s current position. This might be better accomplished by performing targeted keyword searches of project documents with a gender theme in the PO database, or through interviews with Bank staff, particularly those involved in its 2016-2023 gender strategy.**

 Second, while it is clearly highly appropriate to use the gender policy marker for analyzing projects in the CRS database, it would be helpful to know if there are any data on how well this marker captures gender-specific project financing.  Might some projects be missed?  Would there be value, for example, in taking a sample of projects that were not captured by the marker to see if gender was included?

**We have added a few sentences in the discussion, about the fact that *all *three gender databases that we analyse (the PO, DT, and Monitoring Gender Mainstreaming databases) seem to be missing projects. The PO and DT databases have very different projects listed (and only four overlapping projects), despite the fact that they are sorted by gender theme, the Monitoring Gender Mainstreaming database only includes very recent HNP projects, and only one project is listed in all three databases. We also point-out that many projects with a ‘3’ (highest) gender informed indicator rating are not classified as having even a low percentage gender theme, such that there is considerable ambiguity. We suggest in our conclusion that a very valuable follow-up research project will be to take a sample of projects that have a gender informed rating of 1-3, and searching their project documents to see if and in what ways gender objectives and outcomes indicators are addressed. Similarly, it would be very valuable to take all health sector projects with a gender theme in the PO database, and look for references to gender in their project information and appraisal documents (including any gender informed indicator scores), to see how well they match with data reported in the Monitoring Gender Mainstreaming database. Again, however, these valuable projects go beyond the scope of our paper.**

****

**Because our analysis is primarily focused on the Bank’s gender project financing and indicators, we decided only to venture into the OECD gender marker as a basic comparison. We have included a sentence that references criticisms of the OECD gender marker (which, like the Bank gender themes and gender informed indicator is largely self-reported based on largely non-transparent staff appraisals of projects).**

 Third, in Figure 2, the numbers from the Development Topics Database don’t seem right—it starts with n = 90, then 8 were dropped, but then the figure still says 90 (after dropping 8, it should say 82). In addition, we think it would be good to mention the date restriction in figure 2 itself and not just in the methods section. Looking at figure 2 right now, readers may think the search was from January 1, 1985 through December 31, 2017 (the figure says 1985-2017, and this would give 92 projects); the methods section states a narrower date range, i.e. to July 1, 2017 (which yields 90 projects).

**This was entirely our error, and we are grateful to you for catching it. The project numbers are updated in Figure 2 (which is now Figure 3). We have also more clearly added the date restriction in the figure.**

 Fourth, it would be good to update the projects, since the World Bank now lists 102 gender projects: http://www.worldbank.org/en/topic/gender/projects/all.

**We respectfully feel that this update is unnecessary, given that we are following the standard practice of clearly noting when we pulled our data from the database prior to beginning analysis. While it is always ideal to have the most up-to-date data, this would require us to re-do most of our data files, calculations, and graphs. If we do a follow-up study, as suggested in the conclusion, we will certainly use the most up-to-date projects listed.**

Presentation

 The presentation is generally clear.

Interpretation

 The conclusions focus primarily on the shortcomings of the reporting mechanisms and indicators for gender mainstreaming in general.  Gender reporting shortcomings were also a key theme in the CGD study on World Bank gender rhetoric.  It would be valuable, we think, to discuss the concrete concerns with the gender project evaluation criteria moving forward and the conclusions could align more with the specific research question on gender-focused health programs.

**You are absolutely right that we could have done a better job discussing tangible recommendations for gender project (or projects considered gender informed) evaluation criteria. We have restructured our discussion to focus more explicitly on three take-home messages about the Bank’s health portfolio, based on our financial analyses (i.e. transparency and poor correlation between gender themes and indicator scores for the health sector; the focus on middle-income countries and implications for the Bank’s poverty agenda; and poor tracking of health trust funds that may have gender components). We have also added significantly to our conclusion, to discuss specific recommendations about how to improve transparency of gender-focused health programmes and suggested future research about the Bank’s health portfolio.**

 There is one comparison that the authors make that we think may not be a valid one. The authors note “the Bank’s recent claim that 98% of its total lending is gender informed” and then they compare this 98% figure with the proportion of IBRD/IDA financing that is specifically for gender projects.  Is this really an apples to apples comparison?  Being “gender informed” is not the same as financing a gender project.

**Our paper’s other reviewer made a similar point, and it is fair. In our new manuscript version, we have addressed this issue by (1) more clearly stating that we considered projects with a distinct gender theme percentage listed to be gender projects, and more clearly defining what is included in the gender informed indicator rating; (2) clearly distinguishing, throughout the methods and results, the gender projects that we tracked in the PO and DT databases from gender informed projects that we tracked in the Monitoring Gender Mainstreaming database; and (3) comparing the Bank’s 98% gender informed financing claim with *both *the gender project theme commitments and the gender informed indicator scores. When combined with our discussion about transparency and missing projects in each database, as well as our qualifications about our findings due to data quality, we believe that the comparison has become much more valid.**

**Thank you again for taking the time to provide us with such constructive feedback.**
